# Endoscopic transpapillary gallbladder drainage using a novel drill dilator

**DOI:** 10.1055/a-2312-8560

**Published:** 2024-05-29

**Authors:** Jun Noda, Yuichi Takano, Masataka Yamawaki, Tetsushi Azami, Fumitaka Niiya, Fumiya Nishimoto, Masatsugu Nagahama

**Affiliations:** 1 Division of Gastroenterology, Department of Internal Medicine, Showa University Fujigaoka Hospital, Yokohama, Japan

In endoscopic transpapillary gallbladder drainage (ETGBD), device insertion through the cystic duct to the gallbladder is challenging 1 . Even if a guidewire can be placed in the gallbladder, the device cannot be inserted due to stone or inflammation of the cystic duct in some cases 2 3 . We experienced a case in which ETGBD was successfully performed using a novel drill-type dilator ( Fig. 1 ).

**Fig. 1:**
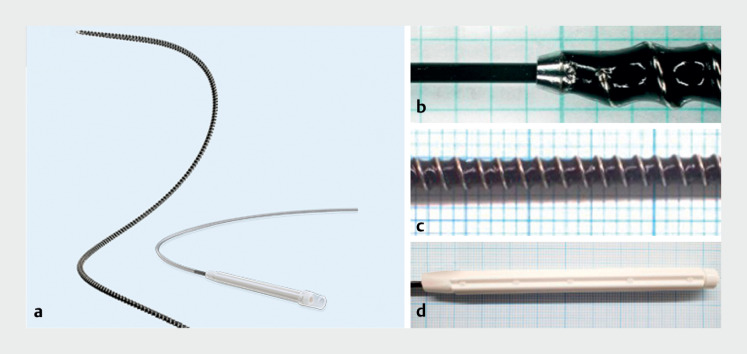
Details of the novel drill-type dilator. **a** The Tornus ES (Olympus Medical Systems, Tokyo, Japan) is a novel spiral drill dilator designed with an outer diameter of 7 Fr. **b** The dilator tip is tapered to match the outer diameter of the guidewire. **c** The shaft is coiled to ensure flexibility. **d** The handle is easy to rotate. Source for Fig. 1 **a** : Olympus Corporation.

A 49-year-old man was brought to the emergency department due to acute cholecystitis and cholangitis, and percutaneous transhepatic gallbladder drainage (PTGBD) was performed. Endoscopic ultrasound revealed a 3-mm stone in the common bile duct. Endoscopic retrograde cholangiopancreatography was performed, and endoscopic stone extraction was performed after endoscopic sphincterotomy. We attempted placement of an endoscopic gallbladder stent (EGBS) until elective cholecystectomy. Although the cystic duct was breached with a guidewire (Visiglide2; Olympus Medical Systems, Tokyo, Japan), the catheter (PR-V614M; Olympus) could not be inserted to the gallbladder due to a stone in the cystic duct ( Fig. 2 **a** ). The novel drill-type dilator was inserted, rotated clockwise, and successfully passed through the cystic duct ( Fig. 2 **b** ). After dilation, a catheter could be inserted into the gallbladder. We switched to a 0.035-inch hard-type guidewire (Revowave Hard; Piolax, Kanagawa, Japan) and a 5 Fr × 32 cm EGBS (IYO-stent; Gadelius, Tokyo, Japan) was successfully placed ( Video 1 ). There were no adverse events associated with the procedure. PTGBD was removed and the patient was scheduled for cholecystectomy.

**Fig. 2:**
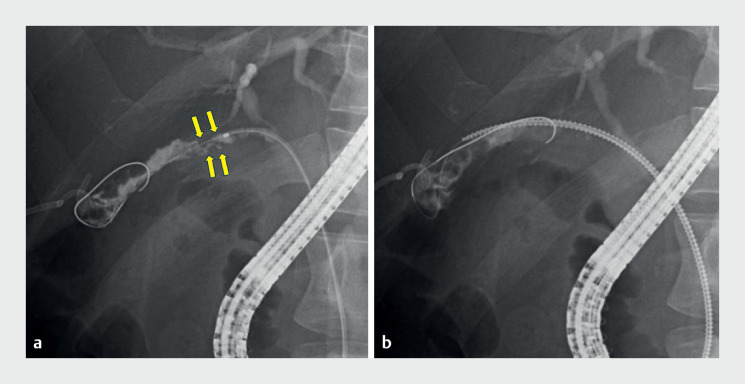
Fluoroscopic images. **a** The catheter could not be inserted to the gallbladder due to a stone (arrows) in the cystic duct. **b** The novel drill-type dilator was carefully inserted and successfully passed through the cystic duct.

We successfully placed a gallbladder stent using the novel drill dilator to breach the cystic duct stricture.Video 1

In cases where device insertion into the gallbladder is difficult, this novel drill-type dilator can be an effective option.

Endoscopy_UCTN_Code_TTT_1AR_2AZ

## Notes

Endoscopy E-Videos https://eref.thieme.de/e-videos
*E-Videos* is an open access online section of the journal *Endoscopy* , reporting on interesting cases and new techniques in gastroenterological endoscopy. All papers include a high-quality video and are published with a Creative Commons CC-BY license. Endoscopy E-Videos qualify for HINARI discounts and waivers and eligibility is automatically checked during the submission process. We grant 100% waivers to articles whose corresponding authors are based in Group A countries and 50% waivers to those who are based in Group B countries as classified by Research4Life (see: https://www.research4life.org/access/eligibility/ ). This section has its own submission website at https://mc.manuscriptcentral.com/e-videos .
